# Identification of priority areas for cholera control, Cameroon

**DOI:** 10.2471/BLT.25.293334

**Published:** 2026-02-03

**Authors:** Armelle Ngomba, Linda Esso, Nicole Fouda Mbarga, Ingrid Kenko, Eric Defo, Nadia Mandeng, Theodore A Tonye, Patricia Mendjime, Chanceline Bilounga, Loic Choupo, Emmanuel Douba, Georges A Etoundi

**Affiliations:** a Faculty of Medicine and Pharmaceutical Sciences, University of Douala, Douala, Cameroon.; b Faculty of Medicine and Biomedical Sciences, University of Yaoundé, Yaoundé, Cameroon.; c Johns Hopkins University, Bloomberg School of Public Health, 615 N. Wolfe Street, Baltimore, MD 21205, United States of America.; d Direction for the Fight against Diseases, Epidemics and Pandemics Control, Ministry of Public Health, Yaoundé, Cameroon.; e Health and Development in Action, Yaoundé, Cameroon.; f Public Health Emergency Operations Centre, Yaoundé, Cameroon.; g World Health Organization Cameroon, Yaoundé, Cameroon.

## Abstract

**Objective:**

To identify priority areas for multisectoral interventions for cholera control in Cameroon.

**Methods:**

We collected data on cholera cases from January 2016 to September 2023 in all 10 regions of Cameroon sourced from the DHIS-2 software, national cholera line lists, situation reports and databases of the *Centre Pasteur du Cameroun* and the National Public Health Laboratory. We entered these data into the Global Task Force on Cholera Control tool to determine a priority index for districts based on four cholera indicators: incidence, mortality, persistence and test positivity. We calculated a vulnerability index based on 12 vulnerability factors. We categorized districts with a priority index ≥ 9 and districts with a priority index < 9 but with ≥ 9 vulnerability factors as priority areas for multisectoral interventions.

**Findings:**

Between 2016 and 2023, Cameroon reported 24 813 suspected cholera cases in nine regions. Of 200 health districts, we identified 48 (24.0%) as priority areas for multisectoral interventions, 35 based on a priority index ≥ 9 and 13 based on vulnerability factors. These priority areas were home to 40.4% (11 488 089/28 433 067) of the country’s population in 2023 and accounted for 91.3% (22 668/24 813) of the cholera cases between 2016 and 2023. Centre, Littoral, South-West and Far North regions account for 85.4% (41/48) of the priority areas for multisectoral interventions.

**Conclusion:**

Identification of priority areas for multisectoral interventions provided evidence for decision-making to enhance cholera preparedness and prevention. The availability of data facilitated this classification, and the ownership and leadership of the main governmental stakeholders were essential.

## Résumé

**Objectif:**

Identifier les zones prioritaires pour les interventions multisectorielles de lutte contre le choléra au Cameroun.

**Méthodes:**

Nous avons recueilli des données sur les cas de choléra entre janvier 2016 et septembre 2023 dans les 10 régions du Cameroun à partir du logiciel DHIS-2, des listes nationales de cas de choléra, des rapports de situation et des bases de données du Centre Pasteur du Cameroun et du Laboratoire national de santé publique. Nous avons saisi ces données dans l’outil du Groupe de travail mondial sur la lutte contre le choléra afin de déterminer un indice de priorité pour les districts sur la base de quatre indicateurs du choléra: incidence, mortalité, persistance et positivité des tests. Nous avons calculé un indice de vulnérabilité sur la base de 12 facteurs de vulnérabilité. Nous avons classé les districts ayant un indice de priorité ≥ 9 et ceux ayant un indice de priorité < 9 mais ≥ 9 facteurs de vulnérabilité comme zones prioritaires pour des interventions multisectorielles.

**Résultats:**

Entre 2016 et 2023, le Cameroun a signalé 24 813 cas suspects de choléra dans neuf régions. Sur 200 districts sanitaires, nous en avons identifié 48 (24,0%) comme zones prioritaires pour des interventions multisectorielles, 35 sur la base d’un indice de priorité ≥ 9 et 13 sur la base de facteurs de vulnérabilité. Ces zones prioritaires abritent 40,4% (11 488 089 / 28 433 067) de la population du pays en 2023 et représentaient 91,3% (22 668 / 24 813) des cas de choléra entre 2016 et 2023. Les régions du Centre, du Littoral, du Sud-Ouest et de l’Extrême-Nord représentent 85,4% (41/48) des zones prioritaires pour les interventions multisectorielles.

**Conclusion:**

L’identification des zones prioritaires pour les interventions multisectorielles a fourni des éléments probants pour la prise de décisions visant à renforcer la préparation et la prévention du choléra. La bonne disponibilité des données a facilité cette classification, et l’appropriation et le leadership des principaux acteurs gouvernementaux ont été essentiels.

## Resumen

**Objetivo:**

Identificar áreas prioritarias para intervenciones multisectoriales destinadas al control del cólera en Camerún.

**Métodos:**

Se recopilaron datos sobre casos de cólera desde enero de 2016 hasta septiembre de 2023 en las 10 regiones de Camerún, procedentes del software DHIS-2, listados nacionales de casos de cólera, informes de situación y bases de datos del Centre Pasteur du Camerún y del Laboratorio Nacional de Salud Pública. Estos datos se introdujeron en la herramienta del Grupo de Trabajo Mundial para el Control del Cólera con el fin de determinar un índice de prioridad por distritos basado en cuatro indicadores de cólera: incidencia, mortalidad, persistencia y positividad de las pruebas. Se calculó un índice de vulnerabilidad basado en 12 factores de vulnerabilidad. Se clasificaron como áreas prioritarias para intervenciones multisectoriales los distritos con un índice de prioridad ≥9 y los distritos con un índice de prioridad <9 pero con ≥9 factores de vulnerabilidad.

**Resultados:**

Entre 2016 y 2023, Camerún notificó 24 813 casos sospechosos de cólera en nueve regiones. De 200 distritos sanitarios, se identificaron 48 (24,0%) como áreas prioritarias para intervenciones multisectoriales: 35 sobre la base de un índice de prioridad ≥9 y 13 sobre la base de factores de vulnerabilidad. Estas áreas prioritarias concentraron el 40,4% (11 488 089/28 433 067) de la población del país en 2023 y representaron el 91,3% (22 668/24 813) de los casos de cólera entre 2016 y 2023. Las regiones Centre, Littoral, South-West y Far North representaron el 85,4% (41/48) de las áreas prioritarias para intervenciones multisectoriales.

**Conclusión:**

La identificación de áreas prioritarias para intervenciones multisectoriales aportó evidencia para la toma de decisiones con miras a reforzar la preparación y la prevención del cólera. La disponibilidad de datos facilitó esta clasificación. Además, la apropiación y el liderazgo de las principales partes interesadas gubernamentales fueron esenciales.

## ملخص

**الغرض:**

تحديد المناطق ذات الأولوية للتدخلات متعددة القطاعات لمكافحة الكوليرا في الكاميرون.

**الطريقة:**

قمنا بجمع بيانات عن حالات الكوليرا من يناير/كانون ثاني 2016 إلى سبتمبر/أيلول 2023 في جميع المناطق العشر في الكاميرون، من خلال برنامج DHIS-2، وقوائم الكوليرا الوطنية، وتقارير الأوضاع، وقواعد بيانات مركز باستور في الكاميرون، والمختبر الوطني للصحة العامة. أدخلنا هذه البيانات في أداة فرقة العمل العالمية لمكافحة الكوليرا، لتحديد مؤشر أولوية للمناطق بناءً على أربعة مؤشرات للكوليرا: معدل الإصابة، والوفيات، واستمرار المرض، ونسبة إيجابية الاختبار. قمنا باحتساب مؤشر نقاط الضعف بناءً على 12 عامل للضعف. قمنا بتصنيف المناطق ذات مؤشر الأولوية ≥ 9، والمناطق ذات مؤشر الأولوية < 9، ولكن مع مؤشر ≥ 9 لعوامل نقاط الضعف مع المناطق ذات أولوية التدخلات متعددة القطاعات.

**النتائج:**

بين عامي 2016 و2023، أعلنت الكاميرون 24813 حالة مشتبه بها بالكوليرا في تسع مناطق. ومن بين 200 منطقة صحية، قمنا بتحديد 48 منطقة (%24.0) كمناطق ذات أولوية للتدخلات متعددة القطاعات، و35 منها بناءً على مؤشر أولوية ≥ 9، و13 بناءً على عوامل نقاط الضعف. وتضم هذه المناطق ذات الأولوية %40.4 (11488089 من أصل 28433067) من سكان البلاد في عام 2023، وشكلت %91.3 (22668 من أصل 24813) من حالات الكوليرا بين عامي 2016 و2023. وتمثل مناطق الوسط، والساحل، والجنوب الغربي، والشمال الأقصى %85.4 (41 من أصل 48) من المناطق ذات الأولوية للتدخلات متعددة القطاعات.

**الاستنتاج:**

نتج عن تحديد المجالات ذات الأولوية للتدخلات متعددة القطاعات، توفير أدلةً لدعم عملية صنع القرار لتعزيز الاستعداد للوقاية. وقد أدى توافر البيانات لتسهيل هذا التصنيف، وكان لملكية وقيادة الأطراف الفاعلة الحكومية تأثير أساسي.

## 摘要

**目的:**

旨在确定喀麦隆需实施霍乱控制多部门干预措施的优先区域。

**方法:**

通过提取 DHIS-2 软件、国家霍乱病例信息列表、情况报告及喀麦隆巴斯德中心和国家公共卫生实验室的数据库，我们收集了喀麦隆 10 个大区从 2016 年 1 月至 2023 年 9 月的霍乱病例数据。我们将这些数据输入全球霍乱控制专题小组的工具，基于四项霍乱指标确定各卫生行政区的优先指数：发病率、死亡率、流行持续性和检测阳性率。基于 12 个脆弱性因素，我们计算出了脆弱性指数。我们将优先指数 ≥ 9 的卫生行政区以及优先指数 < 9 但脆弱性因素有 9 个及以上的卫生行政区归入需实施多部门干预措施的优先区域。

**结果:**

在 2016-2023 年期间，喀麦隆的 9 个大区共报告了 24,813 例疑似霍乱病例。在 200 个卫生行政区中，我们将 48 个（占 24.0%）确定为需实施多部门干预措施的优先区域，其中有 35 个是基于优先指数 ≥ 9 而划定，另外 13 个则是基于脆弱性因素而划定。这些优先区域的人口数占 2023 年全国总人口的 40.4% (11,488,089/28,433,067)，且 2016-2023 年期间这些优先区域的霍乱病例数占总数的 91.3% (22,668/24,813)。中部、滨海、西南和极北四个大区中需实施多部门干预措施的优先区域占总数的 85.4% (41/48)。

**结论:**

确定需实施多部门干预措施的优先区域为决策制定提供了证据，有助于提高霍乱防范和预防能力。相关数据的可获得性确保我们能够顺利开展此次分类工作，而由主要政府利益相关者掌握主导权发挥领导作用则是关键所在。

## Резюме

**Цель:**

Определить приоритетные территории для реализации многосекторальных вмешательств по борьбе с холерой в Камеруне.

**Методы:**

Авторы собрали данные о случаях холеры за период с января 2016 года по сентябрь 2023 года во всех 10 регионах Камеруна из программного обеспечения DHIS-2, национальных линейных списков случаев холеры, ситуационных отчетов, а также баз данных Центра Пастера Камеруна и Национальной лаборатории общественного здравоохранения. Полученные данные были введены в инструмент Глобальной целевой группы по борьбе с холерой для расчета приоритетного индекса для округов на основе четырех показателей холеры: заболеваемости, смертности, устойчивости передачи и доли положительных лабораторных тестов. Также был рассчитан индекс уязвимости на основе 12 факторов уязвимости. Округа с приоритетным индексом от 9 и выше, а также округа с приоритетным индексом менее 9, но с 9 и более факторами уязвимости были отнесены к приоритетным территориям для многосекторальных вмешательств.

**Результаты:**

В период с 2016 по 2023 год в Камеруне было зарегистрировано 24 813 предполагаемых случаев холеры в девяти регионах страны. Из 200 медицинских округов 48 (24,0%) были определены как приоритетные территории для многосекторальных вмешательств: 35 на основании приоритетного индекса от 9 баллов и выше и 13 на основании факторов уязвимости. На этих приоритетных территориях проживает 40,4% населения страны по состоянию на 2023 год (11 488 089 из 28 433 067 человек), на них пришлось 91,3% случаев холеры (22 668 из 24 813), зарегистрированных в период с 2016 по 2023 год. Центральный регион, Литораль, Юго-Запад и Крайний Север страны составляют 85,4% (41 из 48) приоритетных территорий для многосекторальных вмешательств.

**Вывод:**

Определение приоритетных территорий для многосекторальных вмешательств обеспечило доказательную основу для принятия решений, направленных на укрепление готовности к холере и ее профилактики. Наличие данных способствовало проведению данной классификации, а ключевую роль сыграли вовлеченность, ответственность и лидерство основных заинтересованных сторон в органах власти.

## Introduction

Worldwide, cholera trends show an increasing global health challenge. Cholera is a marker of inequity and poverty^1^^,^^2^ with the highest burden in the World Health Organization (WHO) Eastern Mediterranean and African Regions.^3^ The main determinants of cholera are insufficient access to potable water, basic sanitation and hygiene; environmental disasters including floods, droughts, population displacement and conflicts; and poor access to health care.^4^^–^^6^

Cameroon is vulnerable to cholera, with recurrent outbreaks since 1971.^7^ Transmission patterns vary by season, with the infections occurring during the rainy season in the southern regions and the dry season in the northern regions.^7^ Cameroon’s long border with Nigeria,^8^ where cholera is also endemic, increases the risk of cross-border transmission.^9^ As a lower-middle income country with more than 27.9 million people,^10^ Cameroon faces major sanitation and hygiene challenges, with limited access to safe water, inadequate hygiene and poor water treatment.^11^ Ongoing insecurity, humanitarian crises and climate shocks further exacerbate vulnerabilities, resulting in nearly half a million internally displaced people and a similar number of refugees.^12^

From 2018 to 2023, persistent cholera transmission in Cameroon led to the creation of a national cholera plan following the four-phase framework of the Global Task Force on Cholera Control: (i) inception during which countries make political commitments and identify cholera priority areas for multisectoral interventions; (ii) development; (iii) implementation; and (iv) monitoring and reporting of the national cholera plan.^13^^,^^14^ Central to the plan was identifying priority areas for multisectoral interventions, which are zones where conditions favour recurring cholera outbreaks. Given global resource constraints,^3^ focusing efforts on these high-risk areas is important for sustainable cholera control. In this study, we describe the process of identifying these priority areas in Cameroon and the implications for cholera control in the country.

## Methods

### Study design and site

We conducted a retrospective cross-sectional descriptive study in Cameroon, which has 10 regions, 203 health districts and 1819 health areas. We analysed district-level cholera data covering January 2016 to September 2023. A national multisectoral technical team led by the health ministry, supported by WHO, conducted the analysis following guidelines of the Global Task Force on Cholera Control.^15^

### Definitions

In outbreak regions, a suspected cholera case was any patient older than 2 years with acute watery diarrhoea and severe dehydration or death from the diarrhoea. In non-outbreak regions, a suspected cholera case was any case of or death from acute watery diarrhoea. Community cases included any person  2 years and older with profuse acute watery diarrhoea and/or vomiting who remained in the community.^16^ A confirmed case was any person with *Vibrio cholerae* O1 or O139 confirmed by culture or gene amplification.^17^ Epidemiological indicators included incidence (cases per 100 000 person-years); mortality (deaths per 100 000 person-years); persistence (percentage of weeks with  one or more suspected case); and positivity (percentage of suspected cholera cases positive by rapid diagnostic test or culture to detect *V. cholerae*). We defined weekly testing coverage as the percentage of weeks in the study period with at least one suspected case tested by either a rapid diagnostic test or culture. If the weekly testing coverage was greater than 50% in at least 80% of the geographical units, then we considered the representativeness of the cholera laboratory data acceptable and positivity was automatically scored in the tool.^15^

### Data sources

We obtained data from the DHIS-2 software (University of Oslo, Oslo, Norway), national cholera line lists, national and regional situation reports, databases of the *Centre Pasteur du Cameroun* and National Public Health Laboratory, and weekly reports (2016–2025) of the Directorate for the Fight against disease, epidemics and pandemics. We obtained population projections per district for 2016–2023 from the health ministry information unit. We used updated shapefiles for Cameroon’s 10 regions and 203 health districts from the information unit to map priority areas for multisectoral interventions.

### Vulnerability data

We collected a vulnerability data set in September 2023 using KoboCollect (Kobo, Cambridge, United States of America) at regional and district levels. The 12 factors assessed were: (i) lack of access to improved water sources (> 30% of the population); (ii) lack of access to improved sanitation (> 50%); (iii) lack of access to handwashing facilities (> 50% without access); (iv) accessibility of the area; (v) cross-border risk; (vi) travel routes; (vii) overcrowding; (viii) mass gatherings; (ix) presence of high-risk populations (including fishers, internally displaced people and refugees); (x) lack of recent cholera vaccination (> 3 years); (xi) exposure to extreme climate events; and (xii) complex humanitarian emergencies.^15^

### Data analysis

Epidemiological and vulnerability data sets were analysed using the 2023 Excel (Microsoft, Redmond, USA) tool of the Global Task Force on Cholera Control.^15^ This tool automatically calculates a priority index by summing four cholera indicators: incidence, mortality, persistence and test positivity. Indicators were scored from 0 to 3 based on centile thresholds (0: none, 1: < 50th centile, 2: 50th–80th centile and 3: > 80th centile). We considered test positivity representativeness acceptable with 88.5% weekly testing coverage (≥ 50% weekly testing coverage in ≥ 80% of the geographic units). Districts with positivity rates of 0%, ≤ 10%, > 10–30% and > 30% were scored 0, 1, 2 and 3, respectively.^15^

We calculated the priority index as: incidence score + mortality score + persistence score + positivity score.^15^ By summing the 12 binary vulnerability factors (1 point each), we obtained the vulnerability index. The final prioritization score to rank districts was these two indices combined.

### Mapping

We used QGIS version 3.34 (QGIS association, Giswil, Switzerland) and official health ministry shapefiles to map priority areas for multisectoral interventions by district using polygon boundaries.

### Validation and prioritization

A multisectoral validation workshop was held in Mbankomo from 27 November to 1 December 2023, led by the Prime Minister’s Office. Eighty participants from key ministries (health, water and energy, territorial administration, justice, environment, education, finance and local development), technical partners (WHO, United Nations Children’s Fund, United States Agency for International Development, International Federation of Red Cross and Red Crescent Societies, and *Médecins Sans Frontières*), and civil society (*Volontaires pour tous au Cameroun* (VTCAM), *DEMTOU Humanitaire*) attended. The workshop validated results for the priority areas for multisectoral interventions through consensus. Districts with a priority index ≥ 9 were classified as priority areas for multisectoral interventions. Additionally, districts scoring 0–8 but with ≥ 9 vulnerability factors were also classified as priority areas for multisectoral interventions. The threshold of 9 ensured targeted focus and resource optimization.

### Ethical considerations

The study used secondary, anonymized population data, thus ethical clearance was not required. The Directorate for the Fight against disease, epidemics and pandemics approved data use and ensured compliance with national data protection policies, guaranteeing confidentiality and secure data management.

## Results

### Cholera data

Between 2016 and 2023, Cameroon reported 24 813 suspected cholera cases in nine regions with a persistence rate of 51.2% (213 epidemiological weeks out of 416 weeks). Of these cases, 14 036 were tested for *V. cholerae* and 9442 were positive (positivity rate 67.1%): 9620 were tested with a rapid diagnostic test and 7643 (79.4%) were positive; while 4871 were tested by culture and 2018 (41.4%) were positive (Table 1). A total of 680 deaths from cholera were recorded during the same period giving a case fatality rate of 2.7% (680/24 813), a mortality rate of 0.32 per 100 000 inhabitants ((680/215 353 780)  × 100 000) and an incidence of 11.52 per person year ((24 813/215 353 780)  × 100 000).

**Table 1:** Summary of cholera epidemiology, Cameroon, 2016–2023

Year	Population	Suspected cases, no.	Deaths, no.	Case fatality rate, %	Incidence, per 100 000 population	Mortality, per 100 000 population	Total tests, no.^a^	Positive, no. (%)	Tested by rapid diagnostic test	Positive by rapid diagnostic test, no. (%)	Tested by culture	Positive by culture, no. (%)	Weeks with ≥ 1 suspected case, no.	Persistence, %^ b^
2016	24 114 482	78	1	1.3	0.32	0.00	78	78 (100.0)	78	78 (100.0)	1	1 (100.0)	13	25.0
2017	25 935 614	22	NR	0.0	0.08	0.00	22	22 (100.0)	22	22 (100.0)	1	1 (100.0)	9	17.3
2018	26 212 331	1 072	64	6.0	4.09	0.24	426	133 (31.2)	243	87 (35.8)	139	26 (18.7)	23	44.2
2019	26 926 900	906	38	4.2	3.36	0.14	375	298 (79.5)	180	151 (83.9)	78	60 (76.9)	21	40.4
2020	27 730 987	1 879	82	4.4	6.78	0.30	846	523 (61.8)	765	496 (64.8)	76	30 (39.5)	40	76.9
2021	28 729 954	698	24	3.4	2.43	0.08	219	127 (58.0)	72	50 (69.4)	167	74 (44.3)	10	19.2
2022	27 270 447	13 911	272	2.0	51.01	1.00	6 493	3350 (51.6)	2495	1773 (71.1)	4303	1758 (40.9)	52	100.0
2023	28 433 067	6 247	199	3.2	21.97	0.70	5 577	4891 (87.7)	5765	4986 (86.5)	106	68 (64.2)	45	86.5
**Total**	**215 353 782**	**24 813**	**680**	**2.7**	**11.52**	**0.32**	**14 036**	**9422** (**67.1**)	**9620**	**7643** (**79.4**)	**4871**	**2018** (**41.4**)	**213**	**51.2**

NR: not reported.

^a^ Some cases are tested with both a rapid diagnostic test and culture, or only culture, or only rapid diagnostic test depending on the situation (e.g. beginning of an outbreak, availability of reagents, logistics for sample transportation and security).

^b^ % of weeks per year in which cases were detected.

### Evolution and mapping

Between 2016 and 2023, the region of Adamawa did not report any confirmed cholera cases. In 2016, six regions were affected (Centre, East, Far North, Littoral, North and South), with Centre region reporting the most cases (35 without deaths, case fatality rate 0.0%) and Littoral the fewest cases (1 case with 1 death, case fatality rate 100.0%). In 2017, four regions were affected (Centre, Littoral, North and West), with Littoral having the highest number of cases (18 cases with no deaths, case fatality rate 0.0%) and North and West regions the fewest cases (1 case each, with no deaths, case fatality rate 0.0%). In 2018, five regions were affected (Centre, Far North, Littoral, North and South-West). North region reported the most cases (637 cases with 41 deaths, case fatality rate 6.4%) and South-West the least (1 case with no deaths, case fatality rate 0.0%). In 2019, two regions were affected (North and South-West), with the North reporting most cases (536 cases with 22 deaths, case fatality rate 4.1%) and South-West reporting fewer cases (370 cases with 16 deaths, case fatality rate 4.3%). In 2020, four regions were affected (Centre, Littoral, South and South-West), with Littoral reporting the most cases (951 cases with 53 deaths, case fatality rate 5.6%) and Centre the fewest (63 cases without deaths, case fatality rate 0.0%). In 2021, four regions were affected (Centre, Littoral, South and South-West), with South-West reporting the most cases (556 cases with 21 deaths, case fatality rate 3.8%) and Littoral the fewest (30 cases with 1 death, case fatality rate 3.3%). The highest cholera burden during the study period was recorded in 2022, when eight regions were affected (Centre, East, Far North, Littoral, North, West, South and South-West). Littoral reported the highest number (7093 cases with 153 deaths, case fatality rate 2.2%) and East the lowest number (12 cases with 2 deaths, case fatality rate 16.7%). In 2023, six regions were affected (Centre, East, Littoral, West, South and South-West), with Centre reporting the most cases (4653 cases with 155 deaths, case fatality rate 3.3%) and East the fewest (8 cases with 3 deaths, case fatality rate 37.5%).

**Fig. 1:**
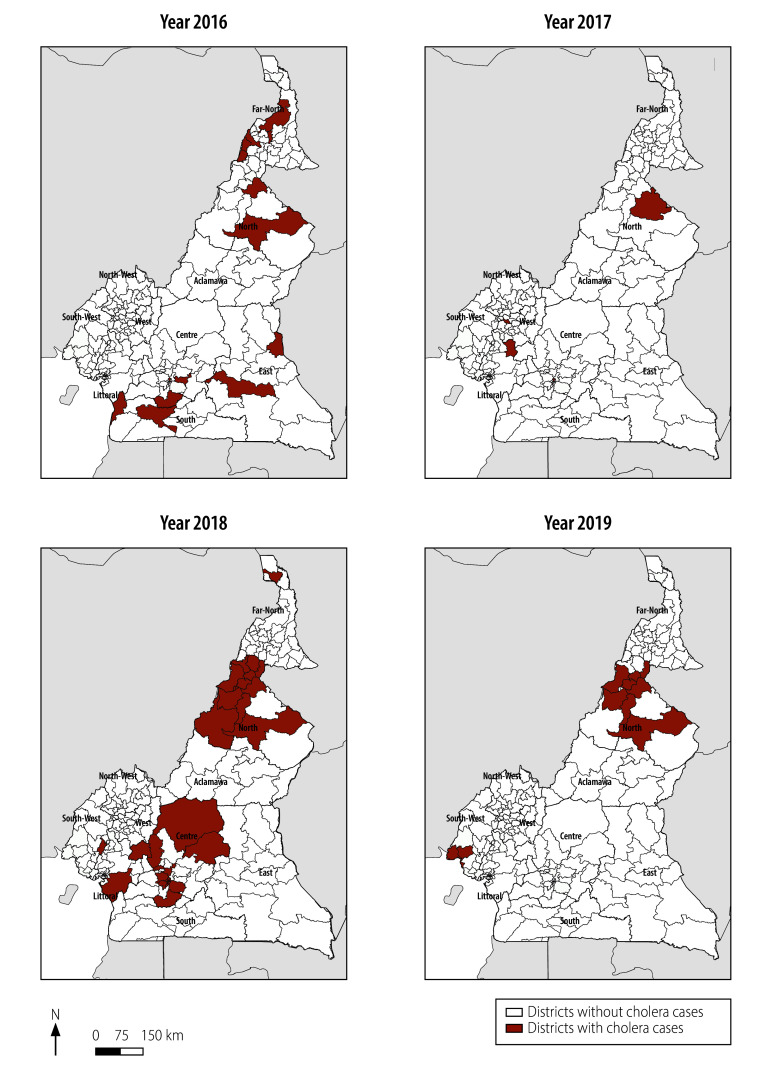
Cholera cases by district and year, Cameroon, 2016–2019

**Fig. 2:**
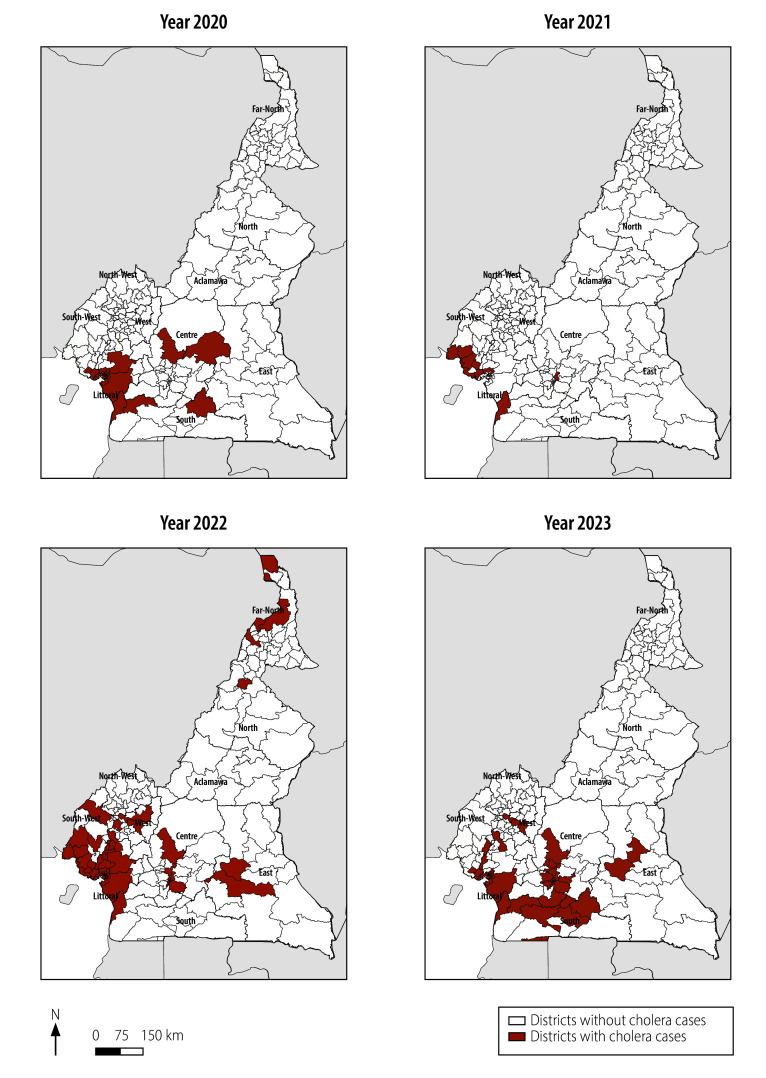
Cholera cases by district and year, Cameroon, 2020–2023

Fig. 1 and Fig. 2 illustrate the trends in cholera cases in Cameroon from 2016 to 2023. The geographical distribution of cases fluctuated over the years, with recurring hotspots in Littoral, Centre and Far North regions.

In 2016, cholera cases were primarily concentrated in Far North, North, East and South regions. By 2017, there was a reduction in the number of cases, with clustering mainly in Littoral and North. In 2018, there was a notable increase in cases, spreading across Centre, Littoral, Far North and West regions (Table 1; Fig. 1 and Fig. 2). Between 2019 and 2021, the distribution of cases fluctuated, with persistent hotspots in Littoral, Centre and Far North regions. In 2022, a significant increase and broader dispersions of cases was seen across these same regions. This regional trend continued in 2023, although no cases were confirmed in Far North (Table 1; Fig. 1 and Fig. 2). Adamawa, East, North-West and West regions did not record any cases between 2016 and 2023.

### Identification of priority areas 

**Fig. 3:**
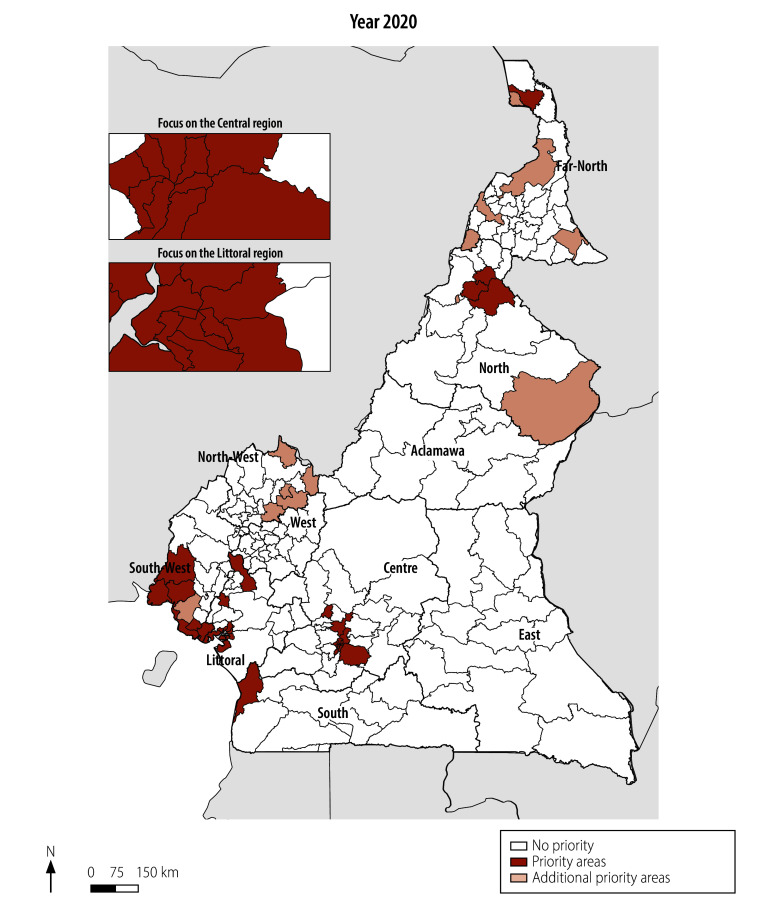
Priority areas for multisectoral interventions for cholera, by district, Cameroon, 2024

Note: The final prioritization integrates four indicators (incidence, mortality, persistence and testing) each assigned specific scores, as well as vulnerability.

In total, we identified 35 health districts as priority areas for multisectoral interventions with a priority index ≥ 9. An additional 13 districts with a priority index < 9 and vulnerability index of ≥ 9 were considered as additional priority areas for multisectoral interventions. Therefore, nationwide, 48 priority areas for multisectoral interventions were identified, with Centre and Littoral regions accounting for 50.0% (24/48) of these priority areas (Table 2 and Table 3). Clustering of these priority areas was noted in four of the 10 regions (Littoral, Centre, South-West and North, in order of the highest number of districts) in Cameroon (Fig. 3).

**Table 2:** Priority areas for multisectoral interventions for cholera based on priority index and vulnerability, Cameroon

Region	No. of districts		
	Priority index ≥ 9	High vulnerability	Total priority areas
Adamawa	0	0	0
Centre	12	0	12
East	0	0	0
Far North	1	5	6
Littoral	12	0	12
North	3	2	5
North-West	0	5	5
West	0	0	0
South	1	0	1
South-West	6	1	7
**Total**	**35**	**13**	**48**

**Table 3:** Final priority areas for multisectoral interventions for cholera, Cameroon, 2024

Region,^a^ health district	Population, no.	Vulnerability index	Incidence score	Persistence score	Mortality score	Test positivity score	Priority index^b^
**Centre**							
Biyem-Assi	422 270	5	2	3	2	3	10
Cité-Verte	472 015	6	2	3	1	3	9
Djoungolo	575 409	7	2	3	2	3	10
Ebebda	23 602	5	2	1	3	3	9
Efoulan	480 107	5	2	3	1	3	9
Mfou	112 210	6	3	2	2	3	10
Mvog-Ada	411 968	4	2	3	1	3	9
Nkolbisson	192 440	6	3	2	2	3	10
Nkolndongo	599 793	8	2	3	2	3	10
Obala	147 550	4	3	3	3	3	12
Odza	442 785	4	2	3	1	3	9
Soa	49 369	5	3	2	2	3	10
**Littoral**							
Bangue	395 315	9	3	3	1	3	10
Boko	373 983	7	3	3	2	3	11
Bonassama	568 369	6	3	3	2	3	11
Cité des palmiers	348 001	4	3	3	1	3	10
Deido	640 608	2	3	3	2	3	11
Japoma	187 006	9	3	3	1	3	10
Logbaba	279 923	4	2	3	1	3	9
Manoka	31 050	9	3	2	3	3	11
Melong	112 946	8	2	2	2	3	9
New-Bell	324 169	8	3	3	3	3	12
Njombe-Penja	59 250	8	3	2	3	3	11
Nylon	462 815	8	3	3	3	3	12
**South-West**							
Bakassi	37 390	7	3	2	3	3	11
Buea	188 293	8	3	2	2	3	10
Ekondo-Titi	61 031	8	3	2	3	3	11
Limbé	215 410	9	3	2	3	3	11
Mbonge	97 999	10	1	1	0	3	5
Mundemba	23 267	8	2	1	3	3	9
Tiko	164 284	8	3	3	3	3	12
**Far North**							
Bourha	90 077	9	1	1	0	3	5
Fotokol	74 704	10	2	2	0	3	7
Makary	144 528	7	2	1	3	3	9
Mokolo	333 305	9	2	1	1	3	7
Mora	328 959	9	1	1	1	3	6
Yagoua	272 701	10	0	0	0	0	0
**North**							
Bibeni	176 023	7	2	2	2	3	9
Garoua	315 119	9	2	2	1	3	8
Golombe	77 146	5	2	2	2	3	9
Pitoa	172 652	8	2	3	3	3	11
Touboro	335 091	9	0	0	0	0	0
**North-West**							
Ako	66 074	9	0	0	0	0	0
Kumbo-East	119 735	9	0	0	0	0	0
Kumbo-West	108 723	9	0	0	0	0	0
Ndop	193 116	10	0	0	0	0	0
Nwa	60 931	9	0	0	0	0	0
**South**							
Kribi	118 579	9	3	3	3	3	12
**Total population**	**11 488 090**	NA	NA	NA	NA	NA	NA

NA: not applicable.

^a^ Ordered by the number of priority areas for multisectoral interventions.

^b^ Calculated as incidence + mortality + persistence + positivity scores.

### Cholera trends, 2024–2025

In 2024, 130 suspected cholera cases were reported, including five confirmed cases: two in Far North region (Maroua 3 district) and three in Centre region (Soa, Djoungolo and Odza districts). In the first half of 2025, 36 suspected cholera cases were reported, 22 of which were positive by rapid diagnostic testing and none confirmed by culture. 

## Discussion

Our study highlights how historic epidemiological data and vulnerability factors can be used to identify priority areas for multisectoral interventions. Using data between 2016 and 2023, 48 health districts in Cameroon were identified as priority areas for multisectoral interventions with an estimated average population at risk of 11 488 089, representing 40.4% of the total population of Cameroon (28 433 067 people in 2023). Our study emphasizes the urgent need to scale up sustainable cholera interventions in these priority areas to reduce this number.

Cholera epidemiology was consistent in Cameroon over the study period and affected a minimum of four regions every year. The northern regions of the country had intense outbreaks between 2018 and 2019, which stopped in 2020. In contrast, from 2020, southern parts of the country became the focus of cholera outbreaks. Cholera persisted between 2018 and 2022 in the South-West region which was almost not affected by cholera outbreaks before 2018.

These trends in cholera outbreaks can be explained by factors including extreme climate events (droughts and floods), a decreased intensity of the humanitarian crisis in Far North region, and increased crises in South-West and North-West regions leading to population displacement towards Littoral, West and Centre regions.^17^ In February 2024, there were more than 1 million internally displaced people and nearly 500 000 refugees and asylum seekers with 1.8 million people in need of sanitation and hygiene services in crisis-affected regions. In Far North region, as of April 2024, the population included 573 263 internally displaced people and refugees from Nigeria, who had moved there due to violence and natural disasters. With only 40% of the population in Far North estimated to have access to improved sanitation and hygiene services, the risk of cholera outbreaks is high in this part of the country.^17^ Violence and fear of attacks from armed groups in South-West and North-West regions have led to regular population displacements. Cameroon is also home to 353 000 refugees from the Central African Republic who mostly live in Eastern region of the country.^17^ A similar dynamic pattern of priority areas for multisectoral interventions has been observed in Ethiopia,^18^^,^^19^ which has faced substantial humanitarian crises associated with climate shocks, disease outbreaks and conflicts in a challenging socioeconomic context.^19^ Continued insecurity and cross-border movements sustain high vulnerability, highlighting the need for regular updates to the priority areas for multisectoral interventions, as recommended by the Global Task Force on Cholera Control, to reflect Cameroon’s dynamic humanitarian and environmental context.^20^

Nearly half of all priority areas for multisectoral interventions are in the urbanized Centre and Littoral regions, which host 34.3% (9 742 188/28 433 067) of the country’s population, including the political and economic capitals. Rapid urbanization, informal settlements and poor town planning have created conditions conducive to cholera transmission, with limited access to sanitation and hygiene services.^21^^–^^24^ There is a serious shortage of water in urban and peri-urban areas in Cameroon.^24^ Rapid urban growth has also led to limited access to health care and the establishment of informal care structures. As such, access to oral rehydration points is limited, resulting in many deaths from cholera. Although cholera deaths declined during the study period, we found a national case fatality rate of 2.7% (680/24 813) which is still higher than the WHO 1% target, indicating challenges in access to health care.^25^ This situation underscores the urgent need for sustained, targeted interventions in high-risk urban and peri-urban priority areas for multisectoral interventions.

One in four districts in Cameroon is at risk of cholera outbreaks and 40.4% (11 488 089/28 433 067) of the total population is at risk of cholera. Thus, Cameroon remains a country with high cholera transmission, which suggests that the country should engage in a control strategy in the national cholera plan rather than an elimination strategy. Similarly, countries such as Burundi, where priority areas for multisectoral interventions represent 25.5% (12/47) of all health districts, would likely opt for a cholera control strategy.^26^ According to the Global Task Force on Cholera Control, to achieve cholera control or elimination, it is important to organize activities around immunization, surveillance, risk communication and community engagement, sanitation and hygiene, and health-system strengthening, and to ensure an effective multisectoral coordination mechanism,^13^^,^^15^ focusing on priority areas for multisectoral interventions. With so many people living at risk of cholera in the priority areas, there are significant financial implications for the cholera control strategy in Cameroon and the need for substantial investment.

The use of oral cholera vaccine for long-term control^27^ in Cameroon would require vaccinating about 11.5 million people with two doses across the 48 priority areas for multisectoral interventions, which poses important cost, logistical and cold-chain challenges. Limited global oral cholera vaccine stockpiles^28^ and co-financing constraints further hinder large-scale preventive campaigns. This global shortage of oral cholera vaccine has prompted a shift to a one-dose strategy.^29^ As such, a phased approach targeting high-risk areas with one dose of the vaccine, combined with strengthened sanitation and hygiene services and behaviour-change interventions, offers a more feasible and sustainable path to cholera control in Cameroon.

The identification and prioritization of priority areas for multisectoral interventions in Cameroon have strengthened cholera control by enabling targeted deployment of rapid diagnostic tests, prepositioning of cholera kits and the development of the 2026–2030 National Cholera Plan. This plan focuses on sanitation and hygiene, surveillance, community engagement, case management, vaccination and governance. Government leadership and ownership was important for ensuring a successful implementation of the exercise. Such cholera control efforts have led to reduced cholera cases since 2024 in Cameroon. Targeting prioritized areas will ensure the optimal use of resources and coordinated multisectoral action in the most vulnerable areas, which aligns with the roadmap of the Global Task Force on Cholera Control.^13^ Without a detailed prioritization process, cholera control efforts risk becoming inefficient and fragmented, with resources spread thin and high-risk areas left vulnerable. This situation can lead to persistent transmission, delayed outbreak response, higher morbidity and mortality, and reduced cost–effectiveness. Ultimately, a lack of prioritization undermines national progress towards cholera elimination and weakens public trust in health interventions.

Despite filling an important evidence gap for decision-making, our study has some limitations, including missing data in newly created districts; incomplete laboratory data before 2018; selection bias in test positivity due to non-systematic testing; variations in surveillance and health-seeking behaviour (notably during the coronavirus disease 2019 pandemic); and effects of administrative changes on trends. These limitations were partially addressed through data imputation, complementary epidemiological indicators, data triangulation with expert validation and harmonized spatial data. Nonetheless, our comprehensive analysis is a first step towards long-term cholera control in Cameroon and beyond.

To conclude, our study sheds light on the use of the Global Task Force on Cholera Control methods to identify priority areas for multisectoral interventions in Cameroon. Our findings provide evidence for public health decision-making to enhance cholera preparedness and prevention in Cameroon and in the African Region by directing interventions to locations where the burden of cholera could be reduced.
